# Frost Bite of the Cornea

**Published:** 1902-01

**Authors:** 


### Frost Bite of the Cornea.

In the *New York Medical Journal,* Dr. E. L. Meirhof reports two cases of frost-bite of the cornea due to the excessive application of cold in the treatment of mild muco purulent conjunctivitis. Both cases were infants less than two weeks old. In both instances after ice cold application had been continued for a few days a slight cloudiness of the cornea developed, which promptly disappeared when warm moist applications were substituted for the cold. He thinks great care should be observed in applying ice cold applications to the eye when the lids are not sufficiently swollen to protect the cornea. R.

